# Advances and challenges in periodontal disease inequalities in Brazil: a population-based comparative analysis, 2010-2023

**DOI:** 10.1590/1980-549720260019.supl.1

**Published:** 2026-07-24

**Authors:** Lívia Valéria Lins e Silva, Bruno Ferraz Barbosa da Costa, Edson Hilan Gomes de Lucena, Yuri Wanderley Cavalcanti, Sabrina Garcia de Aquino

**Affiliations:** I Universidade Federal da Paraíba, Postgraduate Program in Dentistry - João Pessoa (PB), Brazil.; II Universidade Federal da Paraíba, Department of Clinical and Social Dentistry, Health Sciences Center - João Pessoa (PB), Brazil.

**Keywords:** Oral Health, Periodontal Diseases, Health Inequalities, Epidemiological Surveys, Socioeconomic Factors

## Abstract

**Objective:**

To analyze the prevalence of gingival bleeding, dental calculus, and shallow and deep periodontal pockets in the Brazilian National Oral Health Survey (SB Brasil) 2010 and 2023, and to examine their association with socioeconomic determinants.

**Methods:**

Cross-sectional comparative study based on secondary analysis of nationally representative weighted data from SB Brasil. The sample included adolescents (15-19 years), adults (35-44 years), and older adults (65-74 years). The outcomes were gingival bleeding, dental calculus, and shallow and deep periodontal pockets, analyzed as dichotomous variables (presence/absence). Independent variables were sex, income, schooling, and self-reported race/skin color. Multilevel logistic regression models estimated odds ratios and 95% confidence intervals.

**Results:**

Among adolescents, periodontal indicators remained stable between 2010 and 2023. Among adults, lower prevalences of dental calculus and shallow periodontal pockets were observed, with higher odds among men and black and brown individuals for some outcomes in 2023. Among older adults, the prevalence of gingival bleeding and dental calculus was higher in 2023, with higher odds among men and individuals with lower schooling and black race/skin color.

**Conclusion:**

The comparison between SB Brasil 2010 and 2023 reveals heterogeneous changes in periodontal indicators, with stability among adolescents, lower prevalences of dental calculus and shallow periodontal pockets among adults, and higher prevalences of gingival bleeding and dental calculus among older adults in 2023. Differences associated with socioeconomic determinants remained evident, reinforcing the need for continuous monitoring and care strategies throughout life to address persistent inequalities in periodontal health.

## INTRODUCTION

The National Oral Health Policy (PNSB, Política Nacional de Saúde Bucal), launched in 2004 through the “Brasil Sorridente” program, significantly advanced the integration of oral health initiatives into Brazil’s Unified Health System (SUS). Its main achievements include expanding oral health teams within the Family Health Strategy, creating Dental Specialty Centers, which provide specialized diagnosis and treatment for periodontal diseases, and increasing population access to preventive and specialized procedures^1^ ^,^ ^2^. These initiatives aimed to reduce historical disparities in oral health. However, coverage expansion alone is insufficient without addressing structural and contextual determinants^3^ ^,^ ^4^ ^,^ ^5^.

Oral diseases are projected to affect nearly half of the population by 2050, highlighting the urgency of preventive strategies and coordinated health policies. Among them, periodontitis stands out as a highly prevalent chronic condition with a cumulative life-course impact and a major contributor to years lived with disability^6^.

In Brazil, two decades after the implementation of the PNSB, it is crucial to evaluate its outcomes using robust population-based evidence. The national oral health surveys (SB Brasil), conducted in 2010 and 2023, provide representative data on the oral health status of the Brazilian population^4^ ^,^ ^7^. These surveys are part of a nationwide surveillance strategy and reflect methodological improvements over time, particularly in the standardization and quality of periodontal examinations, ensuring greater precision and comparability of periodontal indicators. These surveys allow for the monitoring of changes over time and the direct comparison of relevant clinical indicators^8^ ^,^ ^9^. This comparative approach between independent cross-sectional surveys enables the assessment of changes in disease patterns without implying longitudinal follow-up of individuals, in line with recommendations for standardized oral health surveillance^10^.

In this context, periodontal disease represents a major public health concern due to its high prevalence and significant impact on mastication, nutrition, aesthetics, and quality of life^11^ ^,^ ^12^ ^,^ ^13^ ^,^ ^14^. It is also associated with chronic noncommunicable diseases and contributes to persistent low-grade systemic inflammation in advanced stages, with potential negative implications for general health^10^ ^,^ ^11^ ^,^ ^15^. Despite its relevance, most research and public health interventions still focus on dental caries or tooth loss, and national analyses of periodontal disease remain limited^4^ ^,^ ^6^ ^,^ ^16^. Data from SB Brasil 2010 and 2023 provide a unique opportunity to examine periodontal disease in Brazil across different population groups, particularly in the context of the epidemiological transition marked by increased tooth retention into adulthood and older age, enabling the identification of persistent gaps in access to care^7^ ^,^ ^8^.

Social and demographic factors - such as sex, income, education, and race/skin color - strongly influence the occurrence of periodontal disease^9^ ^,^ ^17^ ^,^ ^18^ ^,^ ^19^. These determinants reflect persistent disparities in access to care and exposure to risk factors^20^ ^,^ ^21^. Such disparities tend to manifest differently across stages of the life course, reflecting cumulative exposures and differential access to preventive and therapeutic services. Thus, temporal comparisons of periodontal indicators can help to examine the persistence of social gradients in oral health, particularly among historically vulnerable groups^3^ ^,^ ^10^, without assuming the elimination of underlying structural disparities.

To date, no nationally representative study has directly compared clinical indicators of periodontal disease within the past 15 years, while simultaneously integrating socioeconomic determinants and age groups. The inclusion of adolescents (15-19 years), adults (35-44 years), and older adults (65-74 years) allows the assessment of periodontal conditions at key stages of the life course, encompassing early manifestations, disease progression, and cumulative effects in later life. Thus, this study aimed to compare the prevalence of gingival bleeding, dental calculus, and shallow and deep periodontal pockets between SB Brasil 2010 and 2023, as well as to examine their associations with socioeconomic determinants, using SB Brasil as the data source for analysis rather than as the objective of the study.

## METHODS

This study consisted of an observational, cross-sectional, comparative analysis, based on a secondary analysis of weighted samples from SB Brasil 2010 and 2023, which are independent, nationally representative surveys^7^. Estimates of periodontal conditions were calculated for adolescents (15-19 years), adults (35-44 years), and older adults (65-74 years) and analyzed across socioeconomic variables.

Data from SB Brasil 2010 and 2023 were retrieved from the Brazilian Ministry of Health website (https://www.gov.br/saude/pt-br/composicao/saps/brasil-sorridente/sb-brasil/dados). The datasets consisted of the complete survey data. Eligible participants were individuals residing in permanent private households in urban areas who belonged to one of the three age groups of interest (15-19, 35-44, and 65-74 years) and had complete periodontal examination data. Individuals classified as Indigenous or yellow were excluded due to the very small number of observations, which could compromise the precision of the estimates. Since the surveys were conducted under a complex sample design, data related to sample weights were used for the appropriate calculation of estimates^6^.

The Community Periodontal Index (CPI) was used as a measurement instrument, and the study outcomes corresponded to four periodontal conditions clinically assessed through the CPI: gingival bleeding, dental calculus, shallow periodontal pockets (probing depth 3.5-5.5 mm), and deep periodontal pockets (probing depth >5.5 mm) across all age groups. All periodontal outcomes were analyzed as dichotomous variables (presence/absence). All examinations were performed by calibrated examiners, who used a World Health Organization probe for clinical assessment. Examiner training and calibration followed standardized protocols of the SB Brasil surveys, as described in the official methodological reports^22^.

Weighted estimates and 95% confidence intervals (CI) were obtained for all age groups in both surveys. Comparisons between SB Brasil 2010 and 2023 were based on differences and overlap of 95%CI, and changes between surveys were described as increases, decreases, or stability in prevalence, avoiding the use of the term “trend” given the comparison of two cross-sectional surveys.

Independent variables consisted of sex (male/female), income (above/below R$ 1,500), years of schooling (low: up to 4 years; medium: 5-8 years; and high: above 8 years), and self-reported race/skin color (white, black, and brown). The terminology race/skin color was adopted in accordance with the classification used in national surveys. Yellow and Indigenous categories were not considered in this study due to their very low frequency. All these socioeconomic variables were self-declared and collected using a questionnaire. Categories were defined according to a previous publication in the literature^22^.

Multiple multilevel logistic regression models were performed using Stata software, version 19. The city level was considered within the multilevel approach and showed minimal influence of approximately 10%, according to the intraclass correlation coefficient. Estimates of statistical association between sex, income, schooling, and race/skin color and periodontal outcomes (gingival bleeding, calculus, shallow pocket, and deep pocket) were obtained for all age groups, considering both the 2010 and 2023 surveys. For all statistical associations, odds ratios (OR) and 95%CI were obtained for each independent variable.

The surveys were analyzed as independent cross-sectional datasets. No longitudinal inference was assumed. The interpretation of social disparities and related issues was intentionally reserved for the Discussion section, in accordance with epidemiological principles and to avoid normative classifications within the analytical methods.

## Data availability statement:

The entire dataset supporting the results of this study is available upon request from the corresponding author. The dataset is not publicly available due to database integration performed by the authors.

## RESULTS

For the 2010 survey, the number of participants was 5,367 adolescents (15-19 years), 9,564 adults (35-44 years), and 7,509 older adults (65-74 years). There were no losses in the calculation of the 2010 survey estimates. For the 2023 survey, the number of participants was 8,041 adolescents, 8,987 adults, and 9,721 older adults. Of these, only participants with complete data were included in the study, resulting in 4,861 adolescents, 6,423 adults, and 6,869 older adults. The main variable with missing data was income.

Table 1 summarizes the socioeconomic characteristics and prevalence of the main periodontal outcomes among participants in the SB Brasil 2010 and 2023 surveys across the age groups 15-19, 35-44, and 65-74 years. Across all age groups, there was a clear shift toward higher income and higher educational attainment between 2010 and 2023. The distribution by sex remained stable, while the proportion of individuals self-identified as brown increased, and that of white decreased. Regarding periodontal outcomes, adolescents showed mostly stable prevalences, adults presented reductions in dental calculus and shallow pockets, and older adults exhibited increases in gingival bleeding and dental calculus.

**Table 1.** Socioeconomic characteristics and prevalence of main periodontal outcomes in epidemiological surveys conducted in 2010 and 2023.

15-19 years	2010			2023		
	Frequency (%)	95%CI (lower-upper)		Frequency (%)	95%CI (lower-upper)	
Sex						
Male	48.29	44.61	51.99	50.07	46.42	53.72
Female	51.71	48.01	55.39	49.93	46.28	53.58
Income						
Lower R$ 1500	67.04	61.59	72.08	30.70	25.81	36.07
Upper R$ 1500	32.96	27.92	38.41	69.30	63.93	74.19
Schooling (years)						
Low (0-4)	4.38	3.05	6.26	1.25	0.71	2.20
Medium (5-8)	30.13	26.18	34.39	8.46	6.74	10.57
High (>8)	65.50	61.43	69.34	90.29	88.05	92.14
Race/skin color						
White	46.90	42.04	51.81	39.95	34.02	46.19
Black	11.28	8.66	14.56	10.97	8.66	13.81
Brown	41.83	37.63	46.14	49.08	43.67	54.50
Gingival bleeding						
No	65.12	59.93	69.97	64.18	59.12	68.95
Yes	34.88	30.03	40.07	35.82	31.05	40.88
Calculus						
No	63.08	58.49	67.44	64.51	59.83	68.94
Yes	36.92	32.56	41.51	35.49	31.06	40.17
Shallow pocket						
No	89.90	87.16	92.11	96.20	94.49	97.40
Yes	10.10	7.89	12.84	3.80	2.60	5.51
Deep pocket						
No	99.18	98.56	99.53	99.60	99.16	99.82
Yes	0.82	0.47	1.44	0.40	0.18	0.84
35-44 years	Frequency (%)	95%CI (lower-upper)		Frequency (%)	95%CI (lower-upper)	
Sex						
Male	36.62	33.52	39.84	33.32	30.32	36.46
Female	63.38	60.16	66.48	66.68	63.54	69.68
Income						
Lower R$ 1500	65.99	61.93	69.83	25.97	21.07	31.56
Upper R$ 1500	34.01	30.17	38.07	74.03	68.44	78.93
Schooling (years)						
Low (0-4)	20.40	17.69	23.41	6.28	4.63	8.47
Medium (5-8)	28.36	25.90	30.95	13.71	11.34	16.47
High (>8)	51.24	47.16	55.30	80.01	76.02	83.49
Race/skin color						
White	50.20	46.62	53.77	43.71	39.12	48.42
Black	11.04	9.56	12.72	13.71	11.22	16.65
Brown	38.76	35.44	42.19	42.58	37.88	47.42
Gingival bleeding						
No	53.46	49.69	57.19	56.24	50.79	61.54
Yes	46.54	42.81	50.31	43.76	38.46	49.21
35-44 years	Frequency (%)	95%CI (lower-upper)		Frequency (%)	95%CI (lower-upper)	
Calculus						
No	34.28	31.86	36.79	44.07	38.92	49.35
Yes	65.72	63.21	68.14	55.93	50.65	61.08
Shallow pocket						
No	72.11	69.30	74.75	86.62	83.15	89.46
Yes	27.89	25.25	30.70	13.38	10.54	16.85
Deep pocket						
No	92.74	90.66	94.39	95.84	91.37	98.04
Yes	7.26	5.61	9.34	4.16	1.96	8.63
65-74 years	Frequency (%)	95%CI (lower-upper)		Frequency (%)	95%CI (lower-upper)	
Sex						
Male	37.65	34.20	41.21	39.63	36.81	42.51
Female	62.35	58.79	65.80	60.37	57.49	63.19
Income						
Lower R$ 1500	69.78	64.98	74.18	22.72	19.71	26.03
Upper R$ 1500	30.22	25.82	35.02	77.28	73.97	80.29
Schooling (years)						
Low (0-4)	62.81	56.29	68.89	40.60	35.87	45.52
Medium (5-8)	21.09	17.49	25.20	26.03	23.25	29.02
High (>8)	16.11	12.77	20.11	33.36	29.95	36.96
Race/skin color						
White	56.02	52.17	59.79	50.49	45.89	55.09
Black	14.06	11.35	17.28	13.21	11.18	15.54
Brown	29.92	26.22	33.90	36.30	31.38	41.52
Gingival bleeding						
No	81.86	78.47	84.81	74.17	70.25	77.74
Yes	18.14	15.19	21.53	25.83	22.26	29.75
Calculus						
No	71.28	68.09	74.27	63.26	59.92	66.48
Yes	28.72	25.73	31.91	36.74	33.52	40.08
Shallow pocket						
No	86.21	83.28	88.69	86.33	83.89	88.44
Yes	13.79	11.31	16.72	13.67	11.56	16.11
Deep pocket						
No	96.83	95.63	97.72	97.98	97.32	98.47
Yes	3.17	2.28	4.37	2.02	1.53	2.68

CI: confidence interval.

Table 2 presents the factors associated with gingival bleeding in the age groups 15-19, 35-44, and 65-74 years in the SB Brasil 2010 and 2023 surveys. Among adolescents (15-19 years), no significant differences were observed between the two periods. In adults (35-44 years), black individuals presented higher odds of gingival bleeding in 2023 compared with white individuals. In the elderly population (65-74 years), women showed lower odds of gingival bleeding than men, whereas individuals with higher educational attainment presented higher odds in 2023.

**Table 2.** Factors associated with gingival bleeding in epidemiological surveys conducted in 2010 and 2023.

15-19 years	2010			2023			Tendency
	OR	95%CI (lower-upper)		OR	95%CI (lower-upper)		
Sex							
Male	ref.			ref.			unchanged (minor disparity)
Female	0.999	0.752	1.327	0.760	0.517	1.117	
Income							
Lower R$ 1500	ref.			ref.			unchanged (minor disparity)
Upper R$ 1500	0.741*	0.533	1.031	0.942	0.654	1.353	
Schooling (years)							
(0-4)	ref.			ref.			unchanged (minor disparity)
Medium (5-8)	0.352	0.390	1.923	0.966	0.272	3.439	
High (>8)	0.202	0.276	1.138	0.942	0.283	3.138	
Race/skin color							
White	ref.			ref.			minor disparity
Black	1.178	0.809	1.714	1.615	0.970	2.690	
Brown	1.445*	1.105	1.890	1.066	0.735	1.546	
35-44 years	OR	95%CI (lower-upper)		OR	95%CI (lower-upper)		Tendency
Sex							
Male	ref.			ref.			unchanged (minor disparity)
Female	1.030	0.852	1.246	0.819	0.633	1.062	
Income							
Lower R$ 1500	ref.			ref.			unchanged (minor disparity)
Upper R$ 1500	0.810	0.623	1.054	0.952	0.725	1.252	
Schooling (years)							
(0-4)	ref.			ref.			minor disparity
Medium (5-8)	1.070	0.842	1.359	2.348	0.988	5.582	
High (>8)	0.739*	0.564	0.968	1.762	0.908	3.420	
Race/skin color							
White	ref.			ref.			major disparity
Black	0.912	0.631	1.319	1.907*	1.010	3.601	
Brown	1.174	0.961	1.435	1.115	0.751	1.657	
65-74 years	OR	95%CI (lower-upper)		OR	95%CI (lower-upper)		Tendency
Sex							
Male	ref.			ref.			major disparity
Female	0.829	0.5902	1.1635	0.642*	0.474	0.870	
Income							
Lower R$ 1500	ref.			ref.			unchanged (minor disparity)
Upper R$ 1500	1.230	0.8590	1.7620	0.976	0.756	1.261	
Schooling (years)							
(0-4)	ref.			ref.			major disparity
Medium (5-8)	0.961	0.6212	1.4882	1.246	0.774	2.006	
High (>8)	0.909	0.4961	1.6652	1.670*	1.177	2.370	
Race/skin color							
White	ref.			ref.			unchanged (minor disparity)
Black	1.313	0.7724	2.2328	1.407	0.978	2.023	
Brown	1.185	0.8109	1.7306	0.877	0.645	1.191	

OR: odds ratio; CI: confidence interval; ref.: reference category.

*Statistically significant (p<0.05) compared with the reference category.

Table 3 indicates the factors associated with the presence of dental calculus in the age groups 15-19, 35-44, and 65-74 years in the SB Brasil 2010 and 2023 surveys. Among adolescents aged 15-19 years, no significant differences were observed in the estimates of dental calculus between 2010 and 2023 across the socioeconomic variables evaluated. Among adults aged 35-44 years, women presented lower odds of dental calculus compared with men (OR=0.618). In the elderly population, men, individuals with higher educational attainment, and black individuals presented a higher prevalence of dental calculus compared with their respective reference groups.

**Table 3.** Factors associated with dental calculus in epidemiological surveys conducted in 2010 and 2023.

15-19 years	2010			2023			Tendency
	OR	95%CI (lower-upper)		OR	95%CI (lower-upper)		
Sex							
Male	ref.			ref.			unchanged (minor disparity)
Female	0.803	0.627	1.029	0.832	0.607	1.141	
Income							
Lower R$ 1500	ref.			ref.			unchanged (minor disparity)
Upper R$ 1500	0.764	0.563	1.037	0.885	0.639	1.225	
Schooling (years)							
(0-4)	ref.			ref.			unchanged (minor disparity)
Medium (5-8)	0.889	0.413	1.916	1.097	0.313	3.851	
High (>8)	0.560	0.282	1.112	0.998	0.307	3.251	
Race/skin color							
White	ref.			ref.			unchanged (minor disparity)
Black	1.096	0.699	1.719	1.297	0.743	2.266	
Brown	1.216	0.878	1.682	1.043	0.667	1.632	
35-44 years	OR	95%CI (lower-upper)		OR	95%CI (lower-upper)		Tendency
Sex							
Male	ref.			ref.			major disparity
Female	0.905	0.703	1.165	0.618*	0.481	0.795	
Income							
Lower R$ 1500	ref.			ref.			unchanged (minor disparity)
Upper R$ 1500	0.804	0.613	1.056	0.957	0.720	1.272	
Schooling (years)							
(0-4)	ref.			ref.			unchanged (minor disparity)
Medium (5-8)	1.111	0.815	1.514	1.853	0.818	4.197	
High (>8)	0.842	0.620	1.143	1.086	0.527	2.238	
Race/skin color							
White	ref.			ref.			unchanged (minor disparity)
Black	0.877	0.642	1.198	1.635	0.879	3.041	
Brown	1.070	0.854	1.341	1.191	0.826	1.717	
65-74 years	OR	95%CI (lower-upper)		OR	95%CI (lower-upper)		Tendency
Sex							
Male	ref.			ref.			major disparity
Female	0.783	0.593	1.035	0.625*	0.501	0.779	
Income							
Lower R$ 1500	ref.			ref.			unchanged (minor disparity)
Upper R$ 1500	1.167	0.856	1.591	0.959	0.754	1.220	
Schooling (years)							
(0-4)	ref.			ref.			major disparity
Medium (5-8)	0.973	0.715	1.324	1.155	0.791	1.686	
High (>8)	1.142	0.754	1.729	1.465*	1.048	2.047	
Race/skin color							
White	ref.			ref.			major disparity
Black	1.318	0.804	2.162	1.479*	1.073	2.039	
Brown	1.191	0.849	1.670	0.936	0.765	1.145	

OR: odds ratio; CI: confidence interval; ref.: reference category.

*Statistically significant (p<0.05) compared with the reference category.

Table 4 highlights the factors associated with the presence of shallow periodontal pockets in the age groups 15-19, 35-44, and 65-74 years in the SB Brasil 2010 and 2023 surveys. Among adolescents (15-19 years), women showed lower odds, whereas individuals with intermediate education and black skin color showed higher odds of shallow pockets compared with their reference categories. In adults (35-44 years), income and education remained stable, but women had lower odds in 2023 compared with men. In the elderly population (65-74 years), a similar pattern was observed, with women showing lower odds in 2023, as well as stability in income, education, and race/skin color.

**Table 4.** Factors associated with shallow pocket in epidemiological surveys conducted in 2010 and 2023.

15-19 years	2010			2023			Tendency
	OR	95%CI (lower-upper)		OR	95%CI (lower-upper)		
Sex							
Male	ref.			ref.			major disparity
Female	0.754	0.537	1.057	0.513*	0.266	0.991	
Income							
Lower R$ 1500)	ref.			ref.			minor disparity
Upper R$ 1500	0.619*	0.393	0.973	1.351	0.611	2.986	
Schooling (years)							
(0-4)	ref.			ref.			unchanged (major disparity)
Medium (5-8)	2.584*	1.189	5.614	5.150*	1.073	24.728	
High (>8)	1.393	0.685	2.835	1.921	0.457	8.083	
Race/skin color							
White)	ref.			ref.			unchanged (major disparity)
Black	2.377*	1.169	4.832	5.415*	1.820	16.112	
Brown	1.107	0.653	1.878	1.192	0.598	2.376	
35-44 years	OR	95%CI (lower-upper)		OR	95%CI (lower-upper)		Tendency
Sex							
Male	ref.			ref.			major disparity
Female	0.905	0.732	1.119	0.640*	0.437	0.936	
Income							
Lower R$ 1500	ref.			ref.			unchanged (minor disparity)
Upper R$ 1500	0.763	0.574	1.015	1.323	0.828	2.115	
Schooling (years)							
(0-4)	ref.			ref.			minor disparity
Medium (5-8)	0.979	0.777	1.234	1.290	0.457	3.638	
High (>8)	0.696*	0.547	0.886	0.802	0.409	1.576	
Race/skin color							
White	ref.			ref.			unchanged (minor disparity)
Black	1.086	0.698	1.688	1.508	0.845	2.691	
Brown	1.187	0.908	1.553	0.999	0.644	1.548	
65-74 years	OR	95%CI (lower-upper)		OR	95%CI (lower-upper)		Tendency
Sex							
Male	ref.			ref.			major disparity
Female	0.721	0.501	1.038	0.682*	0.476	0.978	
Income							
Lower R$ 1500	ref.			ref.			unchanged (minor disparity)
Upper R$ 1500	1.217	0.775	1.913	1.135	0.806	1.599	
Schooling (years)							
(0-4)	ref.			ref.			unchanged (minor disparity)
Medium (5-8)	0.863	0.506	1.469	0.835	0.501	1.392	
High (>8)	0.928	0.510	1.685	1.092	0.669	1.780	
Race/skin color							
White	ref.			ref.			minor disparity
Black	1.328	0.849	2.077	1.189	0.748	1.889	
Brown	1.738*	1.166	2.592	1.060	0.685	1.639	

OR: odds ratio; CI: confidence interval; ref.: reference category.

*Statistically significant (p<0.05) compared with the reference category.

Table 5 presents the factors associated with the presence of deep periodontal pockets in the age groups 15-19, 35-44, and 65-74 years in the SB Brasil 2010 and 2023 surveys. Among adolescents (15-19 years), individuals with higher education in 2010 and those with higher income in 2023 showed lower odds of deep pockets, while no significant changes were found for sex or race/skin color. In adults (35-44 years), women and individuals with medium or high educational attainment showed lower odds compared with men and those with low education. In the elderly population (65-74 years), black individuals presented higher odds of deep pockets in 2023, whereas women consistently showed lower odds across both periods.

**Table 5.** Factors associated with deep pocket in epidemiological surveys conducted in 2010 and 2023.

15-19 years	2010			2023			Tendency
	OR	95%CI (lower-upper)		OR	95%CI (lower-upper)		
Sex							
Male	ref.			ref.			unchanged (minor disparity)
Female	0.697	0.232	2.091	1.170	0.271	5.044	
Income							
Lower R$ 1500	ref.			ref.			major disparity
Upper R$ 1500	0.343	0.073	1.603	0.195*	0.049	0.772	
Schooling (years)							
(0-4)	ref.			ref.			minor disparity
Medium (5-8)	0.809	0.152	4.310	7.905	0.707	88.373	
High (>8)	0.173*	0.032	0.949	3.516	0.343	36.022	
Race/skin color							
White	ref.			ref.			unchanged (minor disparity)
Black	2.563	0.400	16.404	0.631	0.101	3.947	
Brown	1.460	0.425	5.018	0.524	0.148	1.861	
35-44 years	OR	95%CI (lower-upper)		OR	95%CI (lower-upper)		Tendency
Sex							
Male	ref.			ref.			major disparity
Female	0.863	0.483	1.541	0.310*	0.179	0.537	
Income							
Lower R$ 1500	ref.			ref.			unchanged (minor disparity)
Upper R$ 1500	0.998	0.566	1.757	0.453	0.195	1.052	
Schooling (years)							
(0-4)	ref.			ref.			major disparity
Medium (5-8)	1.121	0.795	1.582	0.190*	0.039	0.927	
High (>8)	0.463*	0.236	0.908	0.455*	0.212	0.979	
Race/skin color							
White	ref.			ref.			unchanged (minor disparity)
Black	1.183	0.593	2.359	1.347	0.390	4.655	
Brown	0.983	0.677	1.427	1.615	0.883	2.953	
65-74 years	OR	95%CI (lower-upper)		OR	95%CI (lower-upper)		Tendency
Sex							
Male	ref.			ref.			unchanged (major disparity)
Female	0.434*	0.223	0.845	0.437*	0.251	0.759	
Income							
Lower R$ 1500	ref.			ref.			unchanged (minor disparity)
Upper R$ 1500	0.984	0.525	1.843	0.593	0.310	1.134	
Schooling (years)							
(0-4)	ref.			ref.			unchanged (minor disparity)
Medium (5-8)	1.224	0.516	2.906	1.296	0.667	2.518	
High (>8)	1.330	0.560	3.160	1.722	0.910	3.261	
Race/skin color							
White	ref.			ref.			major disparity
Black	0.497	0.205	1.200	2.493*	1.314	4.728	
Brown	0.934	0.489	1.787	1.702	0.936	3.096	

OR: odds ratio; CI: confidence interval; ref.: reference category.

*Statistically significant (p<0.05) compared with the reference category.

Table 6 summarizes overall patterns for gingival bleeding, dental calculus, and shallow and deep periodontal pockets across the SB Brasil 2010 and 2023 surveys. Among adolescents (15-19 years), indicators remained largely stable, with only shallow pockets showing improvement but persistent differences by sex, education, and race/skin color. In adults (35-44 years), improvements were observed in dental calculus and shallow pockets, but differences by sex and race/skin color emerged for gingival bleeding and deep pockets. In the elderly (65-74 years), gingival bleeding and dental calculus showed worsening patterns, with more pronounced differences by sex, education, and race/skin color, whereas shallow and deep pockets remained relatively stable.

**Table 6.** Summary of tendency, according to age ranges, main periodontal disease outcomes and their associated factors, between epidemiological surveys in 2010 and 2023.

15-19 years	General tendency (2010 to 2023)	Tendency of associated factors (between 2010 and 2023)			
		Sex	Income	Schooling years	Race/skin color
Gingival bleeding	unchanged	unchanged (minor disparity)	unchanged (minor disparity)	unchanged (minor disparity)	minor disparity
Dental calculus	unchanged	unchanged (minor disparity)	unchanged (minor disparity)	unchanged (minor disparity)	unchanged (minor disparity)
Shallow pocket	improved	major disparity	minor disparity	unchanged (major disparity)	unchanged (major disparity)
Deep pocket	unchanged	unchanged (minor disparity)	major disparity	minor disparity	unchanged (minor disparity)
35-44 years	General tendency (2010 to 2023)	Tendency of associated factors (between 2010 and 2023)			
		Sex	Income	Schooling years	Race/skin color
Gingival bleeding	unchanged	unchanged (minor disparity)	unchanged (minor disparity)	minor disparity	major disparity
Dental calculus	improved	major disparity	unchanged (minor disparity)	unchanged (minor disparity)	unchanged (minor disparity)
Shallow pocket	improved	major disparity	unchanged (minor disparity)	minor disparity	unchanged (minor disparity)
Deep pocket	unchanged	major disparity	unchanged (minor disparity)	major disparity	unchanged (minor disparity)
65-74 years	General tendency (2010 to 2023)	Tendency of associated factors (between 2010 and 2023)			
		Sex	Income	Schooling years	Race/skin color
Gingival bleeding	worsened	major disparity	unchanged (minor disparity)	major disparity	unchanged (minor disparity)
Dental calculus	worsened	major disparity	unchanged (minor disparity)	major disparity	major disparity
Shallow pocket	unchanged	major disparity	unchanged (minor disparity)	unchanged (minor disparity)	minor disparity
Deep pocket	unchanged	unchanged (major disparity)	unchanged (minor disparity)	unchanged (minor disparity)	major disparity

Overall, the data indicate that, despite some improvements between 2010 and 2023, social differences in periodontal outcomes persist across age groups, particularly among older adults, with men and black individuals generally presenting worse periodontal conditions.

## DISCUSSION

National epidemiological surveys are essential to guide public policies, especially in Brazil, where reducing oral health inequities remains a central goal of the PNSB^3^ ^,^ ^4^ ^,^ ^19^. The analysis of SB Brasil 2010 and 2023 highlights both advances and persisting challenges related to disparities, helping to direct strategies for populations in situations of vulnerability^7^ ^,^ ^8^. Previous studies confirm that, despite universal coverage, disparities in service use persist among older adults, driven by tooth retention, functional limitations, and barriers to access^5^ ^,^ ^18^. This perspective is particularly relevant for periodontal disease, given its high prevalence, functional and psychosocial impact, and established link to chronic conditions, reinforcing the need for continuous monitoring of periodontal indicators to support public health management^10^ ^,^ ^15^.

Our findings reveal a mixed pattern of stability, improvement, and decline in periodontal outcomes across age groups. Adults showed partial improvement in dental calculus and shallow periodontal pockets; however, major disparities by sex (male) and race/skin color (black) persisted or newly emerged. These findings must be interpreted in light of the chronic nature of periodontal disease, which requires continuous and long-term supportive care, tailored to individual risk, to maintain periodontal stability and prevent recurrence - particularly among populations in situations of vulnerability, for whom discontinuity of care may intensify disease progression^23^ ^,^ ^24^. The worsening of deep periodontal pockets in this group highlights the need for early diagnosis in primary care, systematic periodontal probing, and timely referral to Dental Specialty Centers to prevent disease progression, reduce costs, and strengthen integrated care^10^ ^,^ ^25^. Although some indicators improved - such as reductions in disparities by income, black skin color, and education - these gains were modest. Reducing disparities among older adults remains a major public health challenge^15^. Among older adults, lower service use, higher out-of-pocket costs, and limited insurance coverage reflect cumulative disadvantage^19^.

Adolescents showed stable periodontal indicators and fewer disparities; however, the increase in shallow periodontal pockets may reflect early stages of periodontitis. In addition to declining caries rates, uneven benefits and increases in missing teeth in some Brazilian capitals point to persistent disparities^8^ ^,^ ^9^. Considering that periodontal disease may begin early in life and progress silently, continuous preventive actions and follow-up from adolescence throughout the life course are essential, particularly for populations in situations of vulnerability^8^ ^,^ ^9^ ^,^ ^26^.

Among older adults, gingival bleeding and dental calculus showed worsening trends, accompanied by major disparities according to sex, education, and race/skin color. In this age group, the management of periodontal disease becomes more complex due to its chronic course, the need for ongoing supportive periodontal care, and the accumulation of functional and social limitations across the life course^11^ ^,^ ^14^ ^,^ ^16^ ^,^ ^23^. This pattern may be related to the greater tooth retention observed in SB Brasil 2023^27^, combined with inadequate oral health education, reduced manual dexterity, functional impairments, and limited public policies directed to older adults in situations of vulnerability^7^ ^,^ ^18^. Tailored strategies integrating health promotion, assisted self-care, and specialized services may help reduce risk and disparities in this population^10^ ^,^ ^17^. Sex-related disparities likely reflect behavioral patterns, as women tend to seek care more often, adhere more consistently to preventive practices, and engage more regularly in self-care routines, which may partially explain their better periodontal outcomes^18^ ^,^ ^19^. These findings underscore the importance of gender-responsive oral health policies^20^.

Our findings are consistent with international evidence indicating an increasing absolute burden of periodontitis despite modest declines in age-standardized rates^28^. Persistent inequities by sex, education, and race/skin color reflect structural and contextual determinants and should be interpreted as expressions of long-standing social processes rather than simple temporal variations between surveys^20^. Some longitudinal studies conducted in specific Brazilian contexts have suggested reductions in social disparities in selected oral health outcomes following the implementation of the PNSB, particularly among adult populations; however, such findings are context-dependent and do not necessarily imply the overcoming of structural disparities at the national level^24^. The emerging major disparities by race/skin color and sex, particularly among older adults, align with evidence on racial disparities and the under-recognition of racism as a health determinant^20^. Furthermore, early socioeconomic disparities detected among adolescents and older adults suggest that disparities originate early in life and accumulate across the lifespan^26^. In Brazil, a middle-sociodemographic index country, periodontal diseases show high prevalence and marked regional disparities. Socioeconomic conditions, education, access to dental care, and behavioral factors such as smoking contribute to these disparities. National evidence indicates that less developed regions, especially in the North and Northeast, experience greater difficulty in reducing periodontal disease, reflecting persistent structural constraints in healthcare infrastructure and preventive capacity^21^ ^,^ ^29^.

Despite the expansion of oral health teams and Dental Specialty Centers through the PNSB, such isolated measures remain insufficient. Ensuring sustained and continuous periodontal care within public health systems is critical to prevent disease progression and address social disparities, particularly among populations in situations of vulnerability who face structural barriers to care^5^ ^,^ ^18^ ^,^ ^30^. Latin American consensus statements support the integration of standardized periodontal indicators - such as the CPI and clinical attachment loss - into primary care surveillance, focusing on high-risk groups and addressing both modifiable and structural determinants^10^ ^,^ ^15^ ^,^ ^31^.

Differences between adults and older adults may also reflect cumulative exposure to risk factors, unequal access to preventive services, and broader social determinants^3^. Evidence from SB Brasil 2010 already demonstrated that both individual and contextual characteristics influence periodontal disease^16^ ^,^ ^24^ ^,^ ^32^. Findings from more recent national surveys further indicate that tooth retention, functional limitations, and access barriers account for much of the disparities observed among older adults^18^ ^,^ ^19^. The updated analysis for 2023 suggests that these relationships not only persist but, in some contexts, may have intensified.

These findings carry important implications for oral health equity. Preventive measures should begin early, as disparities emerge during adolescence^8^ ^,^ ^9^ ^,^ ^26^. Adults and older adults require targeted strategies that integrate periodontal care with chronic disease management while addressing barriers related to race/skin color and education^18^ ^,^ ^19^ ^,^ ^20^. Furthermore, national surveys should continue applying standardized indicators and explicitly incorporating social and racial variables to enable the monitoring of oral health disparities, as recommended by Celeste et al. (2023) and Latin American consensus reports^10^ ^,^ ^13^ ^,^ ^15^ ^,^ ^31^.

This study provides nationally representative, population-based estimates of periodontal disease and describes its social distribution within broader social and structural contexts in Brazil. Its comparative cross-sectional design allows the description of differences between survey cycles, but does not support causal inference or definitive conclusions regarding changes in periodontal disease disparities over time.

In conclusion, despite some improvements in specific periodontal indicators between 2010 and 2023, social disparities in periodontal outcomes persist and, in some cases, have become even more pronounced, particularly among older adults. These findings do not indicate the overcoming of structural inequities but rather underscore the persistence of social gradients in periodontal health, reinforcing the need for continuous standardized surveillance and disparities-sensitive oral health policies in Brazil.
