# Supplementary material for: Advances and challenges in periodontal disease inequalities in Brazil: a population-based comparative analysis, 2010-2023
Source: Rev Bras Epidemiol. 2026 Jul 24;29(Suppl 1):e260019supl1. doi: 10.1590/1980-549720260019.supl.1 (PMC13403698; doi:10.1590/1980-549720260019.supl.1)
Supplement: Translation

# Translation: Avanços e desafios nas desigualdades da doença periodontal no Brasil: uma análise comparativa de base populacional, 2010-2023

**DOI:** 10.1590/1980-549720260019.supl.1.2

**Authors:** Lívia Valéria Lins e Silva, Bruno Ferraz Barbosa da Costa, Edson Hilan Gomes de Lucena, Yuri Wanderley Cavalcanti, Sabrina Garcia de Aquino

**Affiliations:** I Universidade Federal da Paraíba, Programa de Pós-Graduação em Odontologia - João Pessoa (PB), Brasil.; II Universidade Federal da Paraíba, Departamento de Odontologia Clínica e Social, Centro de Ciências da Saúde - João Pessoa (PB), Brasil.

**Keywords:** Saúde bucal, Doenças periodontais, Desigualdades de saúde, Inquéritos epidemiológicos, Fatores socioeconômicos

## Abstract

**Objetivo:**

Analisar a prevalência de sangramento gengival, cálculo dentário e bolsas periodontais rasas e profundas na Pesquisa Nacional de Saúde Bucal (SB Brasil) 2010 e 2023, bem como sua associação com determinantes socioeconômicos.

**Métodos:**

Estudo comparativo transversal, baseado em análise secundária de dados ponderados representativos da população do SB Brasil. A amostra incluiu adolescentes (15-19 anos), adultos (35-44 anos) e idosos (65-74 anos). Os desfechos foram sangramento gengival, cálculo dentário e bolsas periodontais rasas e profundas, analisados como variáveis dicotômicas (presença/ausência). As variáveis independentes foram sexo, renda, escolaridade e raça/cor da pele autorreferida. Modelos de regressão logística multinível estimaram odds ratios e respectivos intervalos de confiança de 95%.

**Resultados:**

Entre adolescentes, os indicadores periodontais permaneceram estáveis entre 2010 e 2023. Entre adultos, observaram-se menores prevalências de cálculo dentário e bolsas periodontais rasas, com maiores chances associadas ao sexo masculino e aos indivíduos pretos e pardos para alguns desfechos em 2023. Entre idosos, verificou-se maior prevalência de sangramento gengival e cálculo dentário em 2023, com maiores chances entre homens e indivíduos com menor escolaridade e raça/cor da pele preta.

**Conclusão:**

A comparação entre o SB Brasil 2010 e 2023 revela mudanças heterogêneas nos indicadores periodontais, com estabilidade entre adolescentes, redução de cálculo dentário e bolsas periodontais rasas entre adultos e aumento de sangramento gengival e cálculo dentário entre idosos em 2023. As diferenças associadas aos determinantes socioeconômicos permaneceram evidentes, reforçando a necessidade de monitoramento contínuo e de estratégias de cuidado ao longo do curso de vida para enfrentar desigualdades persistentes em saúde periodontal.

## INTRODUÇÃO

A Política Nacional de Saúde Bucal (PNSB), lançada em 2004 por meio do programa “Brasil Sorridente”, promoveu avanços significativos na integração das ações de saúde bucal ao Sistema Único de Saúde (SUS). Entre seus principais resultados, destacam-se a expansão das equipes de saúde bucal no âmbito da Estratégia Saúde da Família, a criação dos Centros de Especialidades Odontológicas, responsáveis pela oferta de diagnóstico e tratamento especializado de doenças periodontais, e a ampliação do acesso da população a procedimentos preventivos e especializados^1^ ^,^ ^2^. Tais iniciativas tiveram como objetivo a redução das disparidades históricas em saúde bucal. Contudo, a ampliação da cobertura, isoladamente, mostra-se insuficiente quando não acompanhada do enfrentamento dos determinantes estruturais e contextuais^3^ ^,^ ^4^ ^,^ ^5^.

Projeta-se que as doenças bucais afetem quase metade da população mundial até 2050, evidenciando a urgência da implementação de estratégias preventivas e de políticas de saúde coordenadas. Nesse contexto, a periodontite destaca-se como uma condição crônica de elevada prevalência, com impacto cumulativo ao longo do curso de vida e importante contribuição para os anos vividos com incapacidade^6^.

No Brasil, duas décadas após a implementação da PNSB, torna-se essencial avaliar seus resultados com base em evidências robustas de base populacional. Os inquéritos nacionais de saúde bucal (SB Brasil), realizados em 2010 e 2023, fornecem dados representativos sobre o estado de saúde bucal da população brasileira^4^ ^,^ ^7^. Esses inquéritos integram uma estratégia de vigilância em âmbito nacional e evidenciam aprimoramentos metodológicos ao longo do tempo, especialmente no que se refere à padronização e à qualidade dos exames periodontais, o que assegura maior precisão e comparabilidade dos indicadores correspondentes. Ademais, possibilitam o monitoramento de mudanças ao longo do tempo e a comparação direta de indicadores clínicos relevantes^8^ ^,^ ^9^. A abordagem comparativa entre inquéritos transversais independentes permite a avaliação de mudanças nos padrões de doença sem implicar seguimento longitudinal dos indivíduos, em consonância com as recomendações para vigilância padronizada em saúde bucal^10^.

Nesse contexto, a doença periodontal configura-se como um importante problema de saúde pública, em virtude de sua elevada prevalência e do impacto significativo sobre a mastigação, a nutrição, a estética e a qualidade de vida^11^ ^,^ ^12^ ^,^ ^13^ ^,^ ^14^. Ademais, associa-se às doenças crônicas não transmissíveis e, em estágios avançados, contribui para a inflamação sistêmica persistente de baixo grau, com potenciais implicações negativas para a saúde geral^10^ ^,^ ^11^ ^,^ ^15^. Apesar de sua relevância, a maior parte das pesquisas e intervenções em saúde pública ainda se concentra na cárie dentária ou na perda dentária, permanecendo limitadas as análises nacionais sobre a doença periodontal^4^ ^,^ ^6^ ^,^ ^16^. Os dados do SB Brasil 2010 e 2023 constituem uma oportunidade singular para a análise da doença periodontal no Brasil em diferentes grupos populacionais, particularmente no contexto da transição epidemiológica caracterizada pelo aumento da retenção dentária na vida adulta e na velhice. Tal cenário possibilita a identificação de lacunas persistentes no acesso à atenção em saúde bucal^7^ ^,^ ^8^.

Fatores sociais e demográficos - como sexo, renda, escolaridade e raça/cor da pele - exercem influência significativa sobre a ocorrência de doença periodontal^9^ ^,^ ^17^ ^,^ ^18^ ^,^ ^19^. Esses determinantes refletem disparidades persistentes no acesso aos cuidados de saúde e na exposição a fatores de risco^20^ ^,^ ^21^. Tais desigualdades tendem a se manifestar de maneira distinta ao longo das diferentes fases da vida, evidenciando exposições cumulativas e acesso diferenciado a serviços preventivos e terapêuticos. Nesse sentido, comparações temporais de indicadores periodontais podem contribuir para a análise da persistência de gradientes sociais na saúde bucal, especialmente entre grupos historicamente vulneráveis^3^ ^,^ ^10^, sem pressupor a eliminação das disparidades estruturais subjacentes.

Até o momento, não há estudos de abrangência nacional que tenham comparado diretamente os indicadores clínicos da doença periodontal ao longo dos últimos 15 anos, integrando simultaneamente determinantes socioeconômicos e diferentes faixas etárias. A inclusão de adolescentes (15-19 anos), adultos (35-44 anos) e idosos (65-74 anos) possibilita a avaliação das condições periodontais em fases-chave do ciclo de vida, abrangendo manifestações precoces, a progressão da doença e os efeitos cumulativos na idade adulta. Dessa forma, o presente estudo teve como objetivo comparar a prevalência de sangramento gengival, cálculo dentário e bolsas periodontais rasas e profundas entre os levantamentos SB Brasil de 2010 e 2023, bem como analisar suas associações com determinantes socioeconômicos, utilizando o SB Brasil como fonte de dados para análise e não como objeto do estudo.

## MÉTODOS

Este estudo consistiu em uma análise observacional, transversal e comparativa, baseada em análise secundária de amostras ponderadas dos inquéritos SB Brasil 2010 e 2023, os quais consistem em pesquisas independentes e representativas em nível nacional^7^. As estimativas das condições periodontais foram calculadas para adolescentes (15-19 anos), adultos (35-44 anos) e idosos (65-74 anos) e analisadas em relação às variáveis socioeconômicas.

Os dados do SB Brasil 2010 e 2023 foram obtidos a partir do sítio eletrônico do Ministério da Saúde do Brasil (https://www.gov.br/saude/pt-br/composicao/saps/brasil-sorridente/sb-brasil/dados). Os conjuntos de dados correspondiam às bases completas das respectivas pesquisas. Foram elegíveis os participantes residentes em domicílios particulares permanentes localizados em áreas urbanas, pertencentes a uma das três faixas etárias de interesse (15-19, 35-44 e 65-74 anos) e com dados completos de exame periodontal. Indivíduos classificados como indígenas ou amarelos foram excluídos devido ao número reduzido de observações, o que poderia comprometer a precisão das estimativas. Considerando que as pesquisas foram conduzidas com desenho amostral complexo, foram utilizados os pesos amostrais para o cálculo adequado das estimativas^6^.

O Índice Periodontal Comunitário (IPC) foi utilizado como instrumento de mensuração, sendo os desfechos do estudo correspondentes a quatro condições periodontais avaliadas clinicamente por meio desse índice: sangramento gengival, cálculo dentário, bolsas periodontais rasas (profundidade de sondagem de 3,5-5,5 mm) e bolsas periodontais profundas (profundidade de sondagem >5,5 mm), em todas as faixas etárias. Todos os desfechos periodontais foram analisados como variáveis dicotômicas (presença/ausência). Os exames foram realizados por examinadores previamente calibrados, com utilização de sonda periodontal da Organização Mundial da Saúde para avaliação clínica. O treinamento e a calibração dos examinadores seguiram protocolos padronizados das pesquisas SB Brasil, conforme descrito nos relatórios metodológicos oficiais^22^.

Estimativas ponderadas e intervalos de confiança de 95% (IC95%) foram obtidos para todas as faixas etárias em ambas as pesquisas. As comparações entre o SB Brasil 2010 e 2023 foram baseadas nas diferenças e na sobreposição dos IC95%, sendo as variações entre os inquéritos descritas como aumentos, reduções ou estabilidade na prevalência, evitando-se o uso do termo “tendência” em razão da comparação entre dois inquéritos transversais independentes.

As variáveis independentes consistiram em sexo (masculino/feminino), renda (acima/abaixo de R$ 1.500), anos de escolaridade (baixa: até 4 anos; média: 5-8 anos; e alta: acima de 8 anos) e raça/cor da pele autodeclarada (branca, negra e parda). A terminologia raça/cor da pele foi adotada em consonância com a classificação utilizada em pesquisas nacionais. As categorias amarela e indígena não foram incluídas neste estudo em razão da baixa frequência observada. Todas as variáveis socioeconômicas foram autodeclaradas e coletadas por meio de questionário. As categorias foram definidas conforme publicação prévia na literatura^22^.

Foram realizados múltiplos modelos de regressão logística multinível, utilizando o software Stata, versão 19. O nível municipal foi considerado na abordagem multinível e apresentou influência mínima de aproximadamente 10%, de acordo com o coeficiente de correlação intraclasse. Foram estimadas associações entre sexo, renda, escolaridade e raça/cor da pele e os desfechos periodontais (sangramento gengival, cálculo dentário, bolsa periodontal rasa e bolsa periodontal profunda), para todas as faixas etárias, considerando os inquéritos de 2010 e 2023. Para todas as associações, foram obtidas as razões de chances (odds ratio - OR) e os respectivos intervalos de confiança de 95% (IC95%) para cada variável independente.

Os levantamentos foram analisados como conjuntos de dados transversais independentes, não sendo assumidas inferências de natureza longitudinal. A interpretação das disparidades sociais e de questões correlatas foi intencionalmente reservada à seção de Discussão, em conformidade com os princípios epidemiológicos e com vistas a evitar a incorporação de classificações normativas na seção de Métodos analíticos.

## Declaração de disponibilidade de dados:

Todo o conjunto de dados que dá suporte aos resultados deste estudo está disponível mediante solicitação ao autor correspondente. O conjunto de dados não está publicamente disponível devido à integração de banco de dados realizada pelos autores.

## RESULTADOS

Para a pesquisa de 2010, o número de participantes foi de 5.367 adolescentes (15-19 anos), 9.564 adultos (35-44 anos) e 7.509 idosos (65-74 anos). Não foram observadas perdas no cálculo das estimativas referentes a esse inquérito. Na pesquisa de 2023, o número de participantes foi de 8.041 adolescentes, 8.987 adultos e 9.721 idosos. Destes, apenas os participantes com dados completos foram incluídos no estudo, totalizando 4.861 adolescentes, 6.423 adultos e 6.869 idosos. A principal variável com dados faltantes foi renda.

A Tabela 1 resume as características socioeconômicas e a prevalência dos principais desfechos periodontais entre os participantes das pesquisas SB Brasil 2010 e 2023, nas faixas etárias de 15-19, 35-44 e 65-74 anos. Em todas as faixas etárias, observou-se aumento dos níveis de renda e escolaridade entre 2010 e 2023. A distribuição por sexo manteve-se estável, enquanto a proporção de indivíduos que se autodeclararam pardos aumentou e a de brancos diminuiu.

**Tabela 1.** Características socioeconômicas e prevalência dos principais desfechos periodontais em inquéritos epidemiológicos realizados em 2010 e 2023.

15-19 anos	2010			2023		
	Frequência (%)	IC95% (inferior-superior)		Frequência (%)	IC95% (inferior-superior)	
Sexo						
Masculino	48,29	44,61	51,99	50,07	46,42	53,72
Feminino	51,71	48,01	55,39	49,93	46,28	53,58
Renda						
Abaixo R$ 1.500	67,04	61,59	72,08	30,70	25,81	36,07
Acima R$ 1.500	32,96	27,92	38,41	69,30	63,93	74,19
Escolaridade (anos)						
Baixa (0-4)	4,38	3,05	6,26	1,25	0,71	2,20
Média (5-8)	30,13	26,18	34,39	8,46	6,74	10,57
Alta (>8)	65,50	61,43	69,34	90,29	88,05	92,14
Raça/cor da pele						
Branca	46,90	42,04	51,81	39,95	34,02	46,19
Preta	11,28	8,66	14,56	10,97	8,66	13,81
Parda	41,83	37,63	46,14	49,08	43,67	54,50
Sangramento gengival						
Não	65,12	59,93	69,97	64,18	59,12	68,95
Sim	34,88	30,03	40,07	35,82	31,05	40,88
Cálculo dentário						
Não	63,08	58,49	67,44	64,51	59,83	68,94
Sim	36,92	32,56	41,51	35,49	31,06	40,17
Bolsa rasa						
Não	89,90	87,16	92,11	96,20	94,49	97,40
Sim	10,10	7,89	12,84	3,80	2,60	5,51
Bolsa profunda						
Não	99,18	98,56	99,53	99,60	99,16	99,82
Sim	0,82	0,47	1,44	0,40	0,18	0,84
35-44 anos	Frequência (%)	IC95% (inferior-superior)		Frequência (%)	IC95% (inferior-superior)	
Sexo						
Masculino	36,62	33,52	39,84	33,32	30,32	36,46
Feminino	63,38	60,16	66,48	66,68	63,54	69,68
Renda						
Abaixo R$ 1.500	65,99	61,93	69,83	25,97	21,07	31,56
Acima R$ 1.500	34,01	30,17	38,07	74,03	68,44	78,93
Escolaridade (anos)						
Baixa (0-4)	20,40	17,69	23,41	6,28	4,63	8,47
Média (5-8)	28,36	25,90	30,95	13,71	11,34	16,47
Alta (>8)	51,24	47,16	55,30	80,01	76,02	83,49
Raça/cor da pele						
Branca	50,20	46,62	53,77	43,71	39,12	48,42
Preta	11,04	9,56	12,72	13,71	11,22	16,65
Parda	38,76	35,44	42,19	42,58	37,88	47,42
Sangramento gengival						
Não	53,46	49,69	57,19	56,24	50,79	61,54
Sim	46,54	42,81	50,31	43,76	38,46	49,21
35-44 anos	Frequência (%)	IC95% (inferior-superior)		Frequência (%)	IC95% (inferior-superior)	
Cálculo dentário						
Não	34,28	31,86	36,79	44,07	38,92	49,35
Sim	65,72	63,21	68,14	55,93	50,65	61,08
Bolsa rasa						
Não	72,11	69,30	74,75	86,62	83,15	89,46
Sim	27,89	25,25	30,70	13,38	10,54	16,85
Bolsa profunda						
Não	92,74	90,66	94,39	95,84	91,37	98,04
Sim	7,26	5,61	9,34	4,16	1,96	8,63
65-74 anos	Frequência (%)	IC95% (inferior-superior)		Frequência (%)	IC95% (inferior-superior)	
Sexo						
Masculino	37,65	34,20	41,21	39,63	36,81	42,51
Feminino	62,35	58,79	65,80	60,37	57,49	63,19
Renda						
Abaixo R$ 1.500	69,78	64,98	74,18	22,72	19,71	26,03
Acima R$ 1.500	30,22	25,82	35,02	77,28	73,97	80,29
Escolaridade (anos)						
Baixa (0-4)	62,81	56,29	68,89	40,60	35,87	45,52
Média (5-8)	21,09	17,49	25,20	26,03	23,25	29,02
Alta (>8)	16,11	12,77	20,11	33,36	29,95	36,96
Raça/cor da pele						
Branca	56,02	52,17	59,79	50,49	45,89	55,09
Preta	14,06	11,35	17,28	13,21	11,18	15,54
Parda	29,92	26,22	33,90	36,30	31,38	41,52
Sangramento gengival						
Não	81,86	78,47	84,81	74,17	70,25	77,74
Sim	18,14	15,19	21,53	25,83	22,26	29,75
Cálculo dentário						
Não	71,28	68,09	74,27	63,26	59,92	66,48
Sim	28,72	25,73	31,91	36,74	33,52	40,08
Bolsa rasa						
Não	86,21	83,28	88,69	86,33	83,89	88,44
Sim	13,79	11,31	16,72	13,67	11,56	16,11
Bolsa profunda						
Não	96,83	95,63	97,72	97,98	97,32	98,47
Sim	3,17	2,28	4,37	2,02	1,53	2,68

IC: intervalo de confiança.

Em relação aos desfechos periodontais, os adolescentes apresentaram prevalências, em sua maioria, estáveis; os adultos apresentaram redução de cálculo dentário e de bolsas periodontais rasas; e os idosos exibiram aumento de sangramento gengival e de cálculo dentário.

A Tabela 1 resume as características socioeconômicas e a prevalência dos principais desfechos periodontais entre os participantes dos levantamentos SB Brasil 2010 e 2023 nas faixas etárias de 15-19, 35-44 e 65-74 anos. Em todas as faixas etárias, observou-se aumento dos níveis de renda e escolaridade entre 2010 e 2023. A distribuição por sexo manteve-se estável, enquanto a proporção de indivíduos autodeclarados pardos aumentou e a de brancos diminuiu. Em relação aos desfechos periodontais, os adolescentes apresentaram prevalências predominantemente estáveis, os adultos apresentaram redução de cálculo dentário e de bolsas periodontais rasas, e os idosos exibiram aumento de sangramento gengival e de cálculo dentário.

A Tabela 2 apresenta os fatores associados ao sangramento gengival nas faixas etárias de 15-19, 35-44 e 65-74 anos nos levantamentos SB Brasil de 2010 e 2023. Entre os adolescentes (15-19 anos), não foram observadas diferenças significativas entre os dois períodos. Nos adultos (35-44 anos), os indivíduos negros apresentaram maior probabilidade de sangramento gengival em 2023 em comparação com os indivíduos brancos. Na população idosa (65-74 anos), as mulheres apresentaram menor probabilidade de sangramento gengival do que os homens, enquanto os indivíduos com maior escolaridade apresentaram maior probabilidade em 2023.

**Tabela 2.** Fatores associados ao sangramento gengival em inquéritos epidemiológicos realizados em 2010 e 2023.

15-19 anos	2010			2023			Tendência
	OR	IC95% (inferior-superior)		OR	IC95% (inferior-superior)		
Sexo							
Masculino	ref.			ref.			inalterada (pequena disparidade)
Feminino	0,999	0,752	1,327	0,760	0,517	1,117	
Renda							
Abaixo R$ 1.500	ref.			ref.			inalterada (pequena disparidade)
Acima R$ 1.500	0,741*	0,533	1,031	0,942	0,654	1,353	
Escolaridade (anos)							
Baixa (0-4)	ref.			ref.			inalterada (pequena disparidade)
Média (5-8)	0,352	0,390	1,923	0,966	0,272	3,439	
Alta (>8)	0,202	0,276	1,138	0,942	0,283	3,138	
Raça/cor da pele							
Branca	ref.			ref.			pequena disparidade
Preta	1,178	0,809	1,714	1,615	0,970	2,690	
Parda	1,445*	1,105	1,890	1,066	0,735	1,546	
35-44 anos	OR	IC95% (inferior-superior)		OR	IC95% (inferior-superior)		Tendência
Sexo							
Masculino	ref.			ref.			inalterada (pequena disparidade)
Feminino	1,030	0,852	1,246	0,819	0,633	1,062	
Renda							
Abaixo R$ 1.500	ref.			ref.			inalterada (pequena disparidade)
Acima R$ 1.500	0,810	0,623	1,054	0,952	0,725	1,252	
Escolaridade (anos)							
Baixa (0-4)	ref.			ref.			pequena disparidade
Média (5-8)	1,070	0,842	1,359	2,348	0,988	5,582	
Alta (>8)	0,739*	0,564	0,968	1,762	0,908	3,420	
Raça/cor da pele							
Branca	ref.			ref.			grande disparidade
Preta	0,912	0,631	1,319	1,907*	1,010	3,601	
Parda	1,174	0,961	1,435	1,115	0,751	1,657	
65-74 anos	OR	IC95% (inferior-superior)		OR	IC95% (inferior-superior)		Tendência
Sexo							
Masculino	ref.			ref.			grande disparidade
Feminino	0,829	0,5902	1,1635	0,642*	0,474	0,870	
Renda							
Abaixo R$ 1.500	ref.			ref.			inalterada (pequena disparidade)
Acima R$ 1.500	1,230	0,8590	1,7620	0,976	0,756	1,261	
Escolaridade (anos)							
Baixa (0-4)	ref.			ref.			grande disparidade
Média (5-8)	0,961	0,6212	1,4882	1,246	0,774	2,006	
Alta (>8)	0,909	0,4961	1,6652	1,670*	1,177	2,370	
Raça/cor da pele							
Branca	ref.			ref.			inalterada (pequena disparidade)
Preta	1,313	0,7724	2,2328	1,407	0,978	2,023	
Parda	1,185	0,8109	1,7306	0,877	0,645	1,191	

OR: odds ratio; IC: intervalo de confiança; ref.: categoria de referência.

*Estatisticamente significativo (p<0,05) em comparação com a categoria de referência.

A Tabela 3 apresenta os fatores associados à presença de cálculo dentário nas faixas etárias de 15-19, 35-44 e 65-74 anos, nas pesquisas SB Brasil de 2010 e 2023. Entre os adolescentes de 15-19 anos, não foram observadas diferenças estatisticamente significativas nas estimativas de cálculo dentário entre 2010 e 2023 em relação às variáveis socioeconômicas avaliadas. Entre os adultos de 35-44 anos, as mulheres apresentaram menor probabilidade de cálculo dentário quando comparadas aos homens (OR=0,618). Na população idosa, observou-se maior prevalência de cálculo dentário entre homens, indivíduos com maior escolaridade e indivíduos negros, em comparação aos respectivos grupos de referência.

**Tabela 3.** Fatores associados ao cálculo dentário em inquéritos epidemiológicos realizados em 2010 e 2023.

15-19 anos	2010			2023			Tendência
	OR	IC95% (inferior-superior)		OR	IC95% (inferior-superior)		
Sexo							
Masculino	ref.			ref.			inalterada (pequena disparidade)
Feminino	0,803	0,627	1,029	0,832	0,607	1,141	
Renda							
Abaixo R$ 1.500	ref.			ref.			inalterada (pequena disparidade)
Acima R$ 1.500	0,764	0,563	1,037	0,885	0,639	1,225	
Escolaridade (anos)							
Baixa (0-4)	ref.			ref.			inalterada (pequena disparidade)
Média (5-8)	0,889	0,413	1,916	1,097	0,313	3,851	
Alta (>8)	0,560	0,282	1,112	0,998	0,307	3,251	
Raça/cor da pele							
Branca	ref.			ref.			inalterada (pequena disparidade)
Preta	1,096	0,699	1,719	1,297	0,743	2,266	
Parda	1,216	0,878	1,682	1,043	0,667	1,632	
35-44 anos	OR	IC95% (inferior-superior)		OR	IC95% (inferior-superior)		Tendência
Sexo							
Masculino	ref.			ref.			grande disparidade
Feminino	0,905	0,703	1,165	0,618*	0,481	0,795	
Renda							
Abaixo R$ 1.500	ref.			ref.			inalterada (pequena disparidade)
Acima R$ 1.500	0,804	0,613	1,056	0,957	0,720	1,272	
Escolaridade (anos)							
Baixa (0-4)	ref.			ref.			inalterada (pequena disparidade)
Média (5-8)	1,111	0,815	1,514	1,853	0,818	4,197	
Alta (>8)	0,842	0,620	1,143	1,086	0,527	2,238	
Raça/cor da pele							
Branca	ref.			ref.			inalterada (pequena disparidade)
Preta	0,877	0,642	1,198	1,635	0,879	3,041	
Parda	1,070	0,854	1,341	1,191	0,826	1,717	
65-74 anos	OR	IC95% (inferior-superior)		OR	IC95% (inferior-superior)		Tendência
Sexo							
Masculino	ref.			ref.			grande disparidade
Feminino	0,783	0,593	1,035	0,625*	0,501	0,779	
Renda							
Abaixo R$ 1.500	ref.			ref.			inalterada (pequena disparidade)
Acima R$ 1.500	1,167	0,856	1,591	0,959	0,754	1,220	
Escolaridade (anos)							
Baixa (0-4)	ref.			ref.			grande disparidade
Média (5-8)	0,973	0,715	1,324	1,155	0,791	1,686	
Alta (>8)	1,142	0,754	1,729	1,465*	1,048	2,047	
Raça/cor da pele							
Branca	ref.			ref.			grande disparidade
Preta	1,318	0,804	2,162	1,479*	1,073	2,039	
Parda	1,191	0,849	1,670	0,936	0,765	1,145	

OR: odds ratio; IC: intervalo de confiança; ref.: categoria de referência.

*Estatisticamente significativo (p<0,05) em comparação com a categoria de referência.

A Tabela 4 apresenta os fatores associados à presença de bolsas periodontais rasas nas faixas etárias de 15-19, 35-44 e 65-74 anos, nos levantamentos SB Brasil de 2010 e 2023. Entre os adolescentes (15-19 anos), observou-se menor probabilidade de bolsas periodontais rasas entre as mulheres, enquanto indivíduos com escolaridade intermediária e de pele negra apresentaram maior probabilidade em comparação às respectivas categorias de referência. Entre os adultos (35-44 anos), renda e escolaridade mantiveram-se estáveis ao longo do período analisado; entretanto, as mulheres apresentaram menor probabilidade de bolsas periodontais rasas em 2023 quando comparadas aos homens. Na população idosa (65-74 anos), observou-se padrão semelhante, com menor probabilidade entre as mulheres em 2023, além de estabilidade nas associações com renda, escolaridade e raça/cor da pele.

**Tabela 4.** Fatores associados à bolsa periodontal rasa em inquéritos epidemiológicos realizados em 2010 e 2023.

15-19 anos	2010			2023			Tendência
	OR	IC95% (inferior-superior)		OR	IC95% (inferior-superior)		
Sexo							
Masculino	ref.			ref.			grande disparidade
Feminino	0,754	0,537	1,057	0,513*	0,266	0,991	
Renda							
Abaixo R$ 1.500	ref.			ref.			pequena disparidade
Acima R$ 1.500	0,619*	0,393	0,973	1,351	0,611	2,986	
Escolaridade (anos)							
Baixa (0-4)	ref.			ref.			inalterada (grande disparidade)
Média (5-8)	2,584*	1,189	5,614	5,150*	1,073	24,728	
Alta (>8)	1,393	0,685	2,835	1,921	0,457	8,083	
Raça/cor da pele							
Branca	ref.			ref.			inalterada (grande disparidade)
Preta	2,377*	1,169	4,832	5,415*	1,820	16,112	
Parda	1,107	0,653	1,878	1,192	0,598	2,376	
35-44 anos	OR	IC95% (inferior-superior)		OR	IC95% (inferior-superior)		Tendência
Sexo							
Masculino	ref.			ref.			grande disparidade
Feminino	0,905	0,732	1,119	0,640*	0,437	0,936	
Renda							
Abaixo R$ 1.500	ref.			ref.			inalterada (pequena disparidade)
Acima R$ 1.500	0,763	0,574	1,015	1,323	0,828	2,115	
Escolaridade (anos)							
Baixa (0-4)	ref.			ref.			pequena disparidade
Média (5-8)	0,979	0,777	1,234	1,290	0,457	3,638	
Alta (>8)	0,696*	0,547	0,886	0,802	0,409	1,576	
Raça/cor da pele							
Branca	ref.			ref.			inalterada (pequena disparidade)
Preta	1,086	0,698	1,688	1,508	0,845	2,691	
Parda	1,187	0,908	1,553	0,999	0,644	1,548	
65-74 anos	OR	IC95% (inferior-superior)		OR	IC95% (inferior-superior)		Tendência
Sexo							
Masculino	ref.			ref.			grande disparidade
Feminino	0,721	0,501	1,038	0,682*	0,476	0,978	
Renda							
Abaixo R$ 1.500	ref.			ref.			inalterada (pequena disparidade)
Acima R$ 1.500	1,217	0,775	1,913	1,135	0,806	1,599	
Escolaridade (anos)							
Baixa (0-4)	ref.			ref.			inalterada (pequena disparidade)
Média (5-8)	0,863	0,506	1,469	0,835	0,501	1,392	
Alta (>8)	0,928	0,510	1,685	1,092	0,669	1,780	
Raça/cor da pele							
Branca	ref.			ref.			pequena disparidade
Preta	1,328	0,849	2,077	1,189	0,748	1,889	
Parda	1,738*	1,166	2,592	1,060	0,685	1,639	

OR: odds ratio; IC: intervalo de confiança; ref.: categoria de referência.

*Estatisticamente significativo (p<0,05) em comparação com a categoria de referência.

A Tabela 5 apresenta os fatores associados à presença de bolsas periodontais profundas nas faixas etárias de 15-19, 35-44 e 65-74 anos, de acordo com os levantamentos SB Brasil de 2010 e 2023. Entre os adolescentes (15-19 anos), indivíduos com maior escolaridade em 2010 e com maior renda em 2023 apresentaram menor probabilidade de bolsas periodontais profundas, não sendo observadas alterações significativas em relação a sexo ou raça/cor da pele. Nos adultos (35-44 anos), mulheres e indivíduos com escolaridade média ou alta apresentaram menor probabilidade de bolsas periodontais profundas quando comparados aos homens e àqueles com baixa escolaridade. Na população idosa (65-74 anos), indivíduos negros apresentaram maior probabilidade de bolsas periodontais profundas em 2023, enquanto as mulheres apresentaram consistentemente menor probabilidade em ambos os períodos avaliados.

**Tabela 5.** Fatores associados à bolsa periodontal profunda em inquéritos epidemiológicos realizados em 2010 e 2023.

15-19 anos	2010			2023			Tendência
	OR	IC95% (inferior-superior)		OR	IC95% (inferior-superior)		
Sexo							
Masculino	ref.			ref.			inalterada (pequena disparidade)
Feminino	0,697	0,232	2,091	1,170	0,271	5,044	
Renda							
Abaixo R$ 1.500	ref.			ref.			grande disparidade
Acima R$ 1.500	0,343	0,073	1,603	0,195*	0,049	0,772	
Escolaridade (anos)							
Baixa (0-4)	ref.			ref.			pequena disparidade
Média (5-8)	0,809	0,152	4,310	7,905	0,707	88,373	
Alta (>8)	0,173*	0,032	0,949	3,516	0,343	36,022	
Raça/cor da pele							
Branca	ref.			ref.			inalterada (pequena disparidade)
Preta	2,563	0,400	16,404	0,631	0,101	3,947	
Parda	1,460	0,425	5,018	0,524	0,148	1,861	
35-44 anos	OR	IC95% (inferior-superior)		OR	IC95% (inferior-superior)		Tendência
Sexo							
Masculino	ref.			ref.			grande disparidade
Feminino	0,863	0,483	1,541	0,310*	0,179	0,537	
Renda							
Abaixo R$ 1.500	ref.			ref.			inalterada (pequena disparidade)
Acima R$ 1.500	0,998	0,566	1,757	0,453	0,195	1,052	
Escolaridade (anos)							
Baixa (0-4)	ref.			ref.			grande disparidade
Média (5-8)	1,121	0,795	1,582	0,190*	0,039	0,927	
Alta (>8)	0,463*	0,236	0,908	0,455*	0,212	0,979	
Raça/cor da pele							
Branca	ref.			ref.			inalterada (pequena disparidade)
Preta	1,183	0,593	2,359	1,347	0,390	4,655	
Parda	0,983	0,677	1,427	1,615	0,883	2,953	
65-74 anos	OR	IC95% (inferior-superior)		OR	IC95% (inferior-superior)		Tendência
Sexo							
Masculino	ref.			ref.			inalterada (grande disparidade)
Feminino	0,434*	0,223	0,845	0,437*	0,251	0,759	
Renda							
Abaixo R$ 1.500	ref.			ref.			inalterada (pequena disparidade)
Acima R$ 1.500	0,984	0,525	1,843	0,593	0,310	1,134	
Escolaridade (anos)							
Baixa (0-4)	ref.			ref.			inalterada (pequena disparidade)
Média (5-8)	1,224	0,516	2,906	1,296	0,667	2,518	
Alta (>8)	1,330	0,560	3,160	1,722	0,910	3,261	
Raça/cor da pele							
Branca	ref.			ref.			grande disparidade
Preta	0,497	0,205	1,200	2,493*	1,314	4,728	
Parda	0,934	0,489	1,787	1,702	0,936	3,096	

OR: odds ratio; IC: intervalo de confiança; ref.: categoria de referência.

*Estatisticamente significativo (p<0,05) em comparação com a categoria de referência.

A Tabela 6 resume os padrões gerais de sangramento gengival, cálculo dentário e bolsas periodontais rasas e profundas nos levantamentos SB Brasil de 2010 e 2023. Entre os adolescentes (15-19 anos), os indicadores permaneceram, em grande parte, estáveis, com melhora observada apenas para bolsas periodontais rasas, embora tenham sido mantidas diferenças segundo sexo, escolaridade e raça/cor da pele. Nos adultos (35-44 anos), observou-se melhora em relação ao cálculo dentário e às bolsas periodontais rasas; contudo, verificou-se o surgimento de diferenças segundo sexo e raça/cor da pele para sangramento gengival e bolsas periodontais profundas. Na população idosa (65-74 anos), o sangramento gengival e o cálculo dentário apresentaram padrão de piora, com diferenças mais acentuadas segundo sexo, escolaridade e raça/cor da pele, enquanto as bolsas periodontais rasas e profundas permaneceram relativamente estáveis.

**Tabela 6.** Síntese das tendências, segundo faixas etárias, principais desfechos de doença periodontal e seus fatores associados, entre os inquéritos epidemiológicos de 2010 e 2023.

15-19 anos	Tendência geral (2010 a 2023)	Tendência dos fatores associados (entre 2010 e 2023)			
		Sexo	Renda	Escolaridade (anos)	Raça/cor da pele
Sangramento gengival	inalterada	inalterada (pequena disparidade)	inalterada (pequena disparidade)	inalterada (pequena disparidade)	pequena disparidade
Cálculo dentário	inalterada	inalterada (pequena disparidade)	inalterada (pequena disparidade)	inalterada (pequena disparidade)	inalterada (pequena disparidade)
Bolsa rasa	melhora	grande disparidade	pequena disparidade	inalterada (grande disparidade)	inalterada (grande disparidade)
Bolsa profunda	inalterada	inalterada (pequena disparidade)	grande disparidade	pequena disparidade	inalterada (pequena disparidade)
35-44 anos	Tendência geral (2010 a 2023)	Tendência dos fatores associados (entre 2010 e 2023)			
		Sexo	Renda	Escolaridade (anos)	Raça/cor da pele
Sangramento gengival	inalterada	inalterada (pequena disparidade)	inalterada (pequena disparidade)	pequena disparidade	grande disparidade
Cálculo dentário	melhora	grande disparidade	inalterada (pequena disparidade)	inalterada (pequena disparidade)	inalterada (pequena disparidade)
Bolsa rasa	melhora	grande disparidade	inalterada (pequena disparidade)	pequena disparidade	inalterada (pequena disparidade)
Bolsa profunda	inalterada	grande disparidade	inalterada (pequena disparidade)	grande disparidade	inalterada (pequena disparidade)
65-74 anos	Tendência geral (2010 a 2023)	Tendência dos fatores associados (entre 2010 e 2023)		
		Sexo	Renda	Escolaridade (anos)	Raça/cor da pele
Sangramento gengival	piora	grande disparidade	inalterada (pequena disparidade)	grande disparidade	inalterada (pequena disparidade)
Cálculo dentário	piora	grande disparidade	inalterada (pequena disparidade)	grande disparidade	grande disparidade
Bolsa rasa	inalterada	grande disparidade	inalterada (pequena disparidade)	inalterada (pequena disparidade)	pequena disparidade
Bolsa profunda	inalterada	inalterada (grande disparidade)	inalterada (pequena disparidade)	inalterada (pequena disparidade)	grande disparidade

De forma geral, os dados indicam que, apesar de algumas melhorias observadas entre 2010 e 2023, as desigualdades sociais nos desfechos periodontais persistem em todas as faixas etárias, particularmente entre os idosos, com homens e indivíduos negros apresentando, em geral, piores condições periodontais.

## DISCUSSÃO

Inquéritos epidemiológicos nacionais são fundamentais para a orientação de políticas públicas, especialmente no Brasil, onde a redução das desigualdades em saúde bucal permanece como objetivo central da PNSB^3^ ^,^ ^4^ ^,^ ^19^. A análise dos levantamentos SB Brasil 2010 e 2023 evidencia tanto avanços quanto desafios persistentes relacionados às disparidades, contribuindo para o direcionamento de estratégias voltadas a populações em situação de vulnerabilidade^7^ ^,^ ^8^. Estudos prévios confirmam que, apesar da cobertura universal, persistem desigualdades no uso de serviços entre idosos, associadas à retenção dentária, às limitações funcionais e às barreiras de acesso^5^ ^,^ ^18^. Essa perspectiva é particularmente relevante no contexto da doença periodontal, dada sua elevada prevalência, seu impacto funcional e psicossocial e sua associação estabelecida com condições crônicas, reforçando a necessidade de monitoramento contínuo de indicadores periodontais para subsidiar a gestão em saúde pública^10^ ^,^ ^15^.

Nossos resultados revelam um padrão misto de estabilidade, melhora e declínio nos desfechos periodontais entre as diferentes faixas etárias. Entre os adultos, observou-se melhora parcial no cálculo dentário e nas bolsas periodontais rasas; contudo, persistiram ou emergiram importantes disparidades segundo sexo (masculino) e raça/cor da pele (negra). Esses achados devem ser interpretados à luz da natureza crônica da doença periodontal, a qual requer cuidados de suporte contínuos e de longo prazo, adaptados ao risco individual, de modo a manter a estabilidade periodontal e prevenir recorrências - especialmente em populações em situação de vulnerabilidade, nas quais a descontinuidade do tratamento pode intensificar a progressão da doença^23^ ^,^ ^24^. O agravamento das bolsas periodontais profundas nesse grupo evidencia a necessidade de diagnóstico precoce na atenção primária, de sondagem periodontal sistemática e de encaminhamento oportuno aos Centros de Especialidades Odontológicas, com vistas à prevenção da progressão da doença, à redução de custos e ao fortalecimento do cuidado integrado^10^ ^,^ ^25^. Embora alguns indicadores tenham apresentado melhora - como a redução de disparidades segundo renda, raça/cor da pele negra e escolaridade - tais avanços foram modestos. A redução das desigualdades entre adultos mais velhos permanece um importante desafio em saúde pública^15^. Entre os idosos, menor utilização de serviços, maiores custos diretos e cobertura de seguro limitada refletem desvantagens cumulativas^19^.

Os adolescentes apresentaram indicadores periodontais estáveis e menor magnitude de disparidades; entretanto, o aumento de bolsas periodontais rasas pode refletir estágios iniciais da periodontite. Além da redução das taxas de cárie, os benefícios desiguais e o aumento da perda dentária em algumas capitais brasileiras apontam para a persistência de desigualdades^8^ ^,^ ^9^. Considerando que a doença periodontal pode iniciar precocemente e evoluir de forma silenciosa, ações preventivas contínuas e o acompanhamento ao longo do curso de vida, desde a adolescência até a idade avançada, são essenciais, particularmente para populações em situação de vulnerabilidade^8^ ^,^ ^9^ ^,^ ^26^.

Entre os idosos, o sangramento gengival e o cálculo dentário apresentaram tendências de piora, acompanhadas por importantes disparidades segundo sexo, escolaridade e raça/cor da pele. Nessa faixa etária, o manejo da doença periodontal torna-se mais complexo em razão de seu curso crônico, da necessidade de cuidados periodontais contínuos e do acúmulo de limitações funcionais e sociais ao longo da vida^11^ ^,^ ^14^ ^,^ ^16^ ^,^ ^23^. Esse padrão pode estar relacionado à maior retenção dentária observada no SB Brasil 2023^27^, associada à educação insuficiente em saúde bucal, à redução da destreza manual, a comprometimentos funcionais e a políticas públicas ainda limitadas voltadas a idosos em situação de vulnerabilidade^7^ ^,^ ^18^. Estratégias individualizadas que integrem promoção da saúde, apoio ao autocuidado e acesso a serviços especializados podem contribuir para a redução do risco e das desigualdades nessa população^10^ ^,^ ^17^. As disparidades segundo sexo provavelmente refletem padrões comportamentais, uma vez que as mulheres tendem a buscar atendimento com maior frequência, aderir de forma mais consistente às práticas preventivas e manter rotinas de autocuidado mais regulares, o que pode explicar, em parte, seus melhores desfechos periodontais^18^ ^,^ ^19^. Esses achados reforçam a importância de políticas de saúde bucal que considerem as dimensões de gênero^20^.

Nossos achados são consistentes com evidências internacionais que indicam aumento da carga absoluta da periodontite, apesar de reduções modestas nas taxas padronizadas por idade^28^. As desigualdades persistentes segundo sexo, escolaridade e raça/cor da pele refletem determinantes estruturais e contextuais e devem ser interpretadas como expressão de processos sociais de longa duração, e não como simples variações temporais entre inquéritos^20^. Alguns estudos longitudinais conduzidos em contextos específicos no Brasil sugeriram redução de desigualdades sociais em determinados desfechos de saúde bucal após a implementação da PNSB, especialmente entre adultos; contudo, tais achados são dependentes do contexto e não implicam, necessariamente, a superação das desigualdades estruturais em nível nacional^24^. As principais disparidades emergentes segundo raça/cor da pele e sexo, particularmente entre idosos, estão alinhadas a evidências sobre desigualdades raciais e ao sub-reconhecimento do racismo como determinante de saúde^20^. Adicionalmente, as desigualdades socioeconômicas observadas precocemente entre adolescentes e idosos sugerem que tais iniquidades se originam no início da vida e se acumulam ao longo do curso de vida^26^. No Brasil, país de índice sociodemográfico médio, as doenças periodontais apresentam elevada prevalência e marcantes disparidades regionais. Condições socioeconômicas, nível educacional, acesso aos cuidados odontológicos e fatores comportamentais, como o tabagismo, contribuem para essas desigualdades. Evidências nacionais indicam que regiões menos desenvolvidas, especialmente Norte e Nordeste, apresentam maior dificuldade na redução da doença periodontal, refletindo limitações estruturais persistentes na infraestrutura de saúde e na capacidade de prevenção^21^ ^,^ ^29^.

Apesar da expansão das equipes de saúde bucal e dos Centros de Especialidades Odontológicas no âmbito da PNSB, tais medidas, isoladamente, ainda se mostram insuficientes. A garantia de cuidado periodontal contínuo e sustentado nos sistemas públicos de saúde é fundamental para prevenir a progressão da doença e enfrentar as desigualdades sociais, especialmente entre populações em situação de vulnerabilidade que enfrentam barreiras estruturais de acesso aos serviços de saúde^5^ ^,^ ^18^ ^,^ ^30^. Declarações de consenso latino-americanas apoiam a integração de indicadores periodontais padronizados - como o IPC e a perda de inserção clínica - na vigilância da atenção primária, com foco em grupos de maior risco e na abordagem de determinantes tanto modificáveis quanto estruturais^10^ ^,^ ^15^ ^,^ ^31^.

As diferenças observadas entre adultos e idosos podem também refletir a exposição cumulativa a fatores de risco, o acesso desigual a serviços preventivos e determinantes sociais mais amplos^3^. Evidências do SB Brasil 2010 já haviam demonstrado que características individuais e contextuais influenciam a ocorrência da doença periodontal^16^ ^,^ ^24^ ^,^ ^32^. Resultados de pesquisas nacionais mais recentes indicam, ainda, que a retenção dentária, as limitações funcionais e as barreiras de acesso explicam parcela importante das disparidades observadas entre idosos^18^ ^,^ ^19^. A análise atualizada referente a 2023 sugere que essas associações não apenas persistem, mas, em alguns contextos, podem ter se intensificado.

Esses achados têm importantes implicações para a equidade em saúde bucal. Medidas preventivas devem ser iniciadas precocemente, uma vez que as desigualdades se manifestam já na adolescência^8^ ^,^ ^9^ ^,^ ^26^. Adultos e idosos demandam estratégias direcionadas que integrem o cuidado periodontal ao manejo de doenças crônicas, com enfrentamento de barreiras relacionadas à raça/cor da pele e à escolaridade^18^ ^,^ ^19^ ^,^ ^20^. Ademais, pesquisas nacionais devem manter a utilização de indicadores padronizados e incorporar de forma explícita variáveis sociais e raciais, de modo a possibilitar o monitoramento das desigualdades em saúde bucal, conforme recomendado por Celeste et al. (2023) e por relatórios de consenso latino-americanos^10^ ^,^ ^13^ ^,^ ^15^ ^,^ ^31^.

Este estudo fornece estimativas populacionais representativas em nível nacional da doença periodontal e descreve sua distribuição social em contextos sociais e estruturais mais amplos no Brasil. Seu delineamento transversal comparativo permite a descrição de diferenças entre os ciclos de inquérito, não sendo possível sustentar inferências causais ou conclusões definitivas acerca de mudanças nas desigualdades da doença periodontal ao longo do tempo.

Em conclusão, apesar de algumas melhorias em indicadores periodontais específicos entre 2010 e 2023, as disparidades sociais nos resultados periodontais persistem e, em alguns casos, tornaram-se ainda mais acentuadas, particularmente entre os idosos. Esses achados não indicam a superação das desigualdades estruturais, mas sim ressaltam a persistência dos gradientes sociais na saúde periodontal, reforçando a necessidade de vigilância padronizada contínua e de políticas de saúde bucal sensíveis às disparidades no Brasil.
